# Live-cell imaging of ER-PM contact architecture by a novel TIRFM approach reveals extension of junctions in response to store-operated Ca^2+^-entry

**DOI:** 10.1038/srep35656

**Published:** 2016-10-19

**Authors:** Michael Poteser, Gerd Leitinger, Elisabeth Pritz, Dieter Platzer, Irene Frischauf, Christoph Romanin, Klaus Groschner

**Affiliations:** 1 Institute of Biophysics, Medical University of Graz, Harrachgasse 21/4, 8010 Graz, Austria; 2 Institute of Cell Biology, Histology and Embryology Research Unit “Electron Microscopic Techniques”, Medical University of Graz, Harrachgasse 21/7, 8010 Graz, Austria; 3 Institute of Biophysics, Johannes Kepler University of Linz, Austria, Gruberstrasse 40, 4020 Linz, Austria.

## Abstract

Nanometer-spaced appositions between endoplasmic reticulum and plasma membrane (ER-PM junctions) stabilized by membrane-joining protein complexes are critically involved in cellular Ca^2+^-handling and lipid trafficking. ER-PM junctional architecture and plasticity associated with inter-membrane communication are as yet barely understood. Here, we introduce a method to precisely characterize ER-PM junction morphology and dynamics with high temporal resolution and minimal disturbance of junctional intermembrane communication. We show that expression of soluble cytosolic fluorophores in combination with TIRFM enables to delineate ER and PM distance in the range of 10–150 nm. Live-cell imaging of sub-plasmalemmal structures in RBL-2H3 mast cells by this method, designated as fluorescence density mapping (FDM), revealed profound dynamics of ER-PM contact sites in response to store-depletion. We report the existence of a Ca^2+^-dependent process that expands the junctional ER to enlarge its contact surface with the PM, thereby promoting and stabilizing STIM1-Orai1 competent ER-PM junctions.

Communication of the endoplasmic reticulum (ER) with other organelles is typically mediated by specialized contact sites (junctions) displaying a membrane nano-architecture that enables efficient exchange of substrates and information. Junctional contact between the endoplasmic reticulum and the plasma membrane is essential for cellular lipid- and Ca^2+^ homeostasis and involves several membrane-joining protein complexes as well as their dynamic assembly and disassembly. While the molecular principles of inter-membrane communication and transport within the junctional space are still incompletely understood, a significant gain in knowledge on ER-PM junctional Ca^2+^ handling has been obtained by the discovery of the STIM-Orai machinery^1^,^2^,^3^,^4^. Several lines of evidence suggest that depletion of internal Ca^2+^-stores and the associated increase in cytosolic Ca^2+^ may impact on junction architecture and function^5^,^6^,^7^. This view is supported by recently gained insights into the junctional role of Ca^2+^-dependent molecules that bridge the ER-PM junctional gap such as extended synaptotagmins (E-Syts)^8^.

Despite an increasing awareness of the (patho)physiological significance of ER-PM junctions and their architectural plasticity, suitable methods for live-cell fluorescence imaging of dynamic morphological changes in these nano-structures have not been developed. This important experimental advance is so far hindered by the lack of fluorescent probes, which reliably report junctional morphology without interfering with architecture and function.

Here we demonstrate a novel approach for live-cell fluorescence imaging of morphology and dynamics of ER-PM junctional structures using total internal reflection microscopy (TIRFM). TIRFM allows for visualization of fluorophores located within or in close proximity to the surface-adherent plasma membrane at high spatial and temporal resolution. We demonstrate that expression of GFP variants with fairly homogenous cytosolic distribution enables imaging of the sub-plasmalemmal topology of organelles on the basis of alterations in thickness of the excited fluorophore layer. Thus, organelle structures within a distance of less than 200 nm of the plasma membrane become visible as darker areas within the bright evanescent field. From the fluorescence intensity, which corresponds strictly with the local fluorophore layer thickness, an intensity map can be constructed that provides information on the contact site shape, area and organelle-PM distance. As the method is based on the reduction of overall cytosolic fluorophore density by local non-fluorescent sub-plasmalemmal structures, we designated this method as fluorescence density mapping (FDM).

In a first application, we utilized FDM in combination with common TIRFM and TIRFM-FRET to investigate the store depletion-induced dynamics of ER-PM junctions in RBL-2H3 mast cells. These experiments provided the first direct observation of SOCE-associated remodeling of ER-PM junctions in living rat mast cells and indicate a tight link between junctional Ca^2+^ handling and architectural dynamics.

## Results

### Theoretical basis of fluorescence density mapping (FDM)

**Figure 1:**
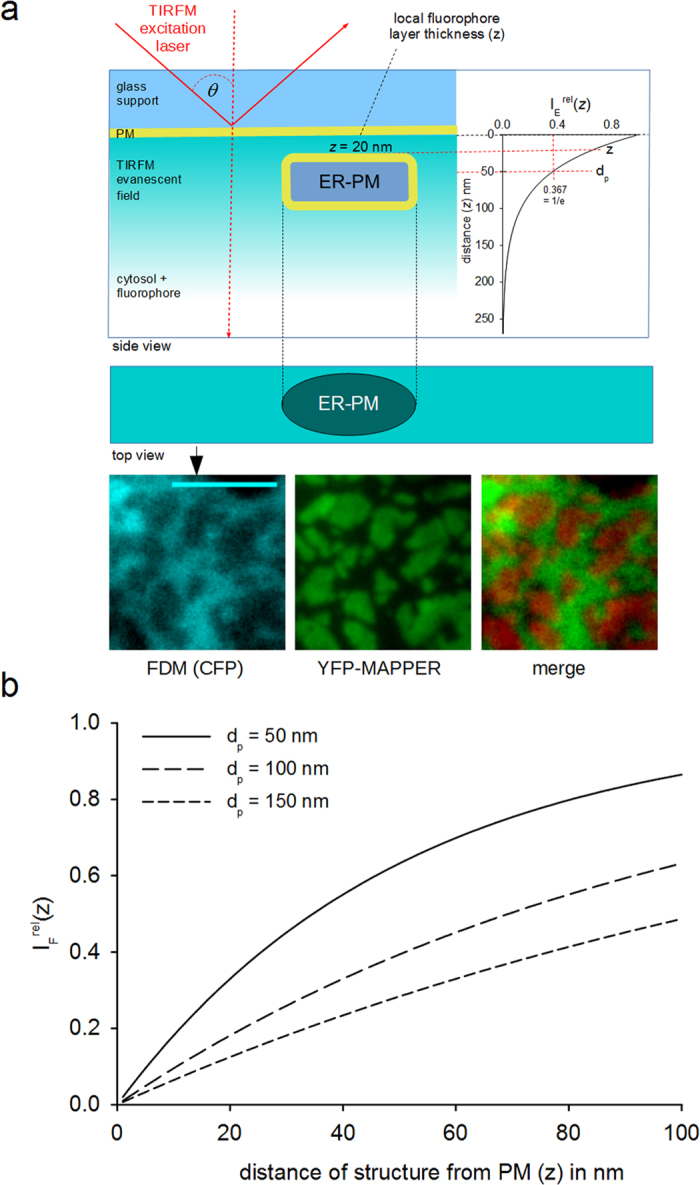
Fluorescence density mapping (FDM). (**a**) Illustration of the theoretical concept: TIRFM illumination exclusively excites fluorophores within the surface-adherent region of the adherent cell by an evanescent wave. Heterologously expressed GFP variants display homogenous distribution in the cytosol. The thickness of the fluorophore-containing cytosolic layer (z) within the evanescent field is limited and determined by the incident angle (*θ*) of the laser excitation light. As the overall emission intensity depends on the local thickness of the excited fluorophore layer, any sub-plasmalemmal structure, intruding this layer will reduce the intensity at this section of the evanescent field. Consequently, non-fluorescent cellular structures within the evanescent field can be discerned as dark regions in FDM. The inserted graph shows the dependence of excitation intensity (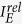, relative to its maximum at the plasma membrane) on z at a given penetration depth (d_p_ = 50 nm; indicated). Bottom: ER-PM junctions in a RBL-2H3 cell visualized by FDM (CFP, cyan) and YFP-MAPPER (green), a STIM1-based marker of ER-PM junctions. (**b**) Dependence of relative fluorescence emission intensity (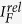, local intensity relative to a maximal value determined in a non-junctional reference point) on the distance (z) between objects intruding the evanescent field and the PM at different penetration depths (d_p_), as calculated by equation 1. Steepness of the curve and, thus, (z-axis) resolution depend on the penetration depth d_p_ of the evanescent field.

Soluble cellular fluorophors with homogenous cytosolic distribution provide a continuous fluorescence within the TIRFM image of a cell’s surface-adherent membrane footprint. Based on the principles of Beer’s law, any non-fluorescent object residing in the membrane-near, excited fluorophore layer will cause a local reduction in overall fluorescence emission, as schematically depicted in Fig. 1a. The relative reduction depends on the proximity of the structure from the PM (z) and the given evanescent wave penetration depth (d_p_). Fluorescence intensity will decline exponentially with the structure approaching the PM, as mathematically described in detail by the equations 3, 4, 5, 6 in the material and methods section.

To obtain an estimate of the object (ER)-PM distance (z), the actual experimental observation variable is the fluorescence intensity at the position of the object in relation to the maximum fluorescence intensity obtained by the evanescent field (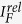) at a given penetration depth (d_p_), as recorded at a position without subplasmalemmal structures:


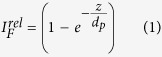


or, transformed to obtain the ER-PM distance z:


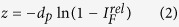


Adjusting d_p_ (by changing TIRF-angle *θ*) allows optimization of the resolution to the depth range of interest (see Fig. 1b). In all our experiments, the TIRFM angle was set to achieve a minimal penetration depth, which is ~50 nm in our experimental setup. Note, that the actual extension of the evanescent field exceeds d_p_ and may induce a (limited) background fluorescence from cellular areas below the structure of interest (residual background).

### ER-PM contacts in resting and store-depleted RBL-2H3 mast cells

**Figure 2:**
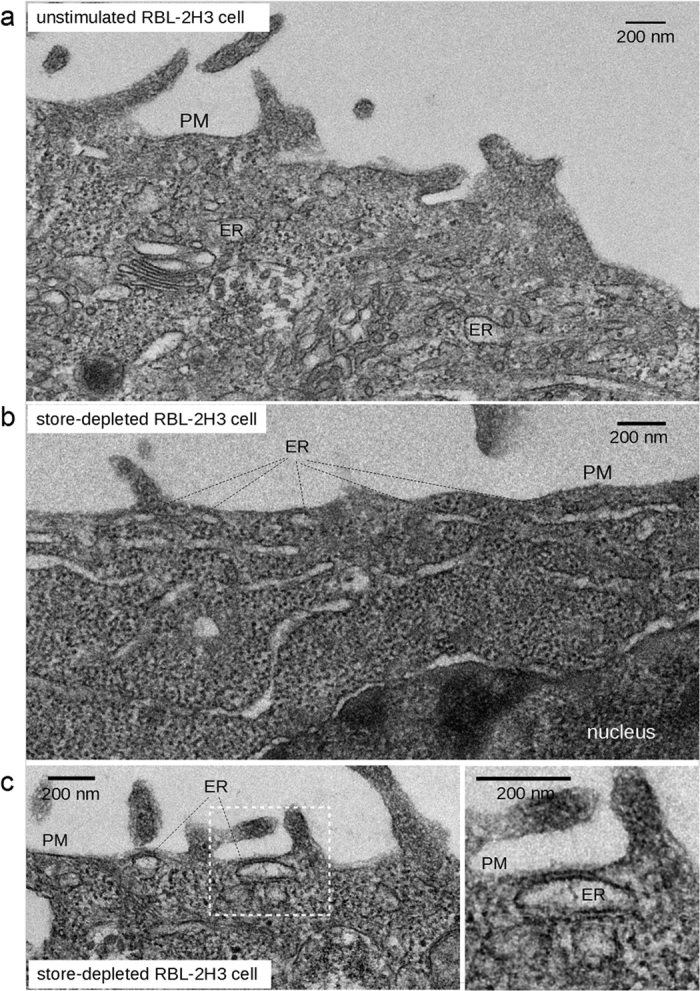
TEM micrographs of RBL-2H3 mast cells. (**a**) TEM micrograph of a cortical section of a non-stimulated RBL-2H3 cell. Portions of the ER are present in the cortical region at variable distances from the PM. (**b**) Cortical ER in a store-depleted RBL-2H3 cell. (**c**) Left: Cortical region of a store depleted RBL-2H3 cell showing ER-PM junctions. Right: Detail of left image as indicated by white box, showing an extended ER-PM junction (>100 nm) with an inter-membrane distance of ~20 nm. ER: endoplasmatic reticulum, PM: plasma membrane. The mean lateral extension of the junctional ER, as represented in EM micrographs, was determined to be 207 ± 23 nm and the mean inter-membrane distance (z) 76 ± 7 nm (S.E.M.), as determined from 20 junctions found in 7 stimulated cells (store-depleted with 5 μM TG for 5 min in Ca^2+^-free buffer followed by 10 min re-addition of Ca^2+^-containing buffer).

For a first cellular application of FDM we choose RBL-2H3 mast cells. This cell type expresses a well characterized form of ER-PM junctions that serve local Ca^2+^ signaling involving the highly Ca^2+^-selective PM ion-channel Orai1, which is physically linked to the ER membrane spanning Ca^2+^-sensor stromal interaction molecule (STIM1)^9^,^10^. STIM-Orai communication is the molecular basis of store-operated calcium entry (SOCE). To gain a first overview on architecture and abundance of ER-PM contact sites in this cell type, sub-plasmalemmal regions in resting and store-depleted RBL-2H3 cells were analyzed by transmission electron microscopy (TEM) (Fig. 2).

We found portions of (smooth) ER in the cortical region of both, resting and store-depleted mast cells.

RBL-2H3 cells displayed smooth ER in the cortical region with no indications of polarized^11^ distribution. In resting cells, the cortical ER was mainly localized within a distance of >100–300 nm from the PM, and only occasionally ER was found at a distance that allows for junctional contact to the PM (<50 nm). However, after store-depletion, well-defined large ER structures were observed abundantly in close proximity of the PM, frequently well-aligned to the PM in a distance of ~15–20 nm (Fig. 2b). Control experiments with vehicle (0.05% DMSO) did not promote the formation of ER-PM junctions (data not shown). In our study, these TEM results provided a first indication for SOCE-induced increases in the extent of junctional ER regions.

### ER-PM junctions visualized by FDM and ER markers

**Figure 3:**
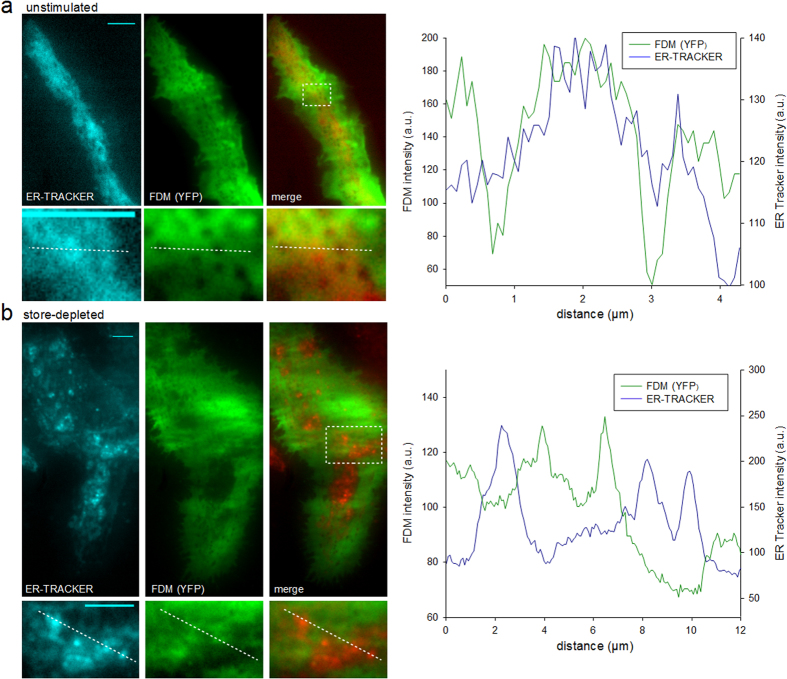
ER-Tracker and FDM identify ER-PM junctions in TIRFM. (**a**) Top, left: TIRFM micrographs of a resting RBL-2H3 cell, labeled with ER-Tracker (glibenglamide BODIPY TR, cyan, left), expressing cytosolic YFP (green, right) and a color overlay image (merge, right). Below: Magnified details of the area indicated in top panel. Right: Intensity line-scans of ER-Tracker fluorescence (blue) and FDM intensities (green) as indicated by the dotted line in detail image. (**b**) Top, left: TIRFM micrographs of a stimulated (5 μM TG for 5 min in Ca^2+^-free buffer solution and 10 min re-addition of Ca^2+^-containing buffer) RBL-2H3 cell, labeled with ER-Tracker (cyan, left), expressing cytosolic YFP (green, right) and a color overlay image (merge, right). Below: Magnified details of the area indicated in top panel. Right: Intensity line-scans of ER-Tracker fluorescence (blue) and FDM intensities (green) as indicated by the dotted line in detail image. Bars = 5 μm.

To test the suitability of FDM for visualization and characterization of ER-PM junction morphology in RBL cells, we first combined FDM with TIRFM of well-established ER fluorescence markers (ER-Tracker, DsRed-ER). Figure 3 shows TIRF images of RBL cells loaded with ER-Tracker to detect ER membranes within the evanescent field and expressing cytosolic GFP as FDM reporter. In basal, unstimulated conditions, most FDM minima were highly spherical, mostly mobile and lacked corresponding ER tracker fluorescence, indicating sub-plasmalemmal vesicles as predominant structures detected by FDM (Fig. 3a).

By contrast, after store depletion with thapsigargin (5 μM TG for 5 min in Ca^2+^-free buffer solution and 10 min re-addition of Ca^2+^-containing buffer) the vast majority of FDM recorded intensity minima were non-spherical, immobile and overlapped with maxima in ER-Tracker fluorescence (Fig. 3b). Mean intensity in FDM minima was 52% ± 0.8% (S.E.M, 4 cells, n = 1029 junctions) of non-junctional cytosolic reference intensity, corresponding to an estimated mean ER-PM distance of ~37 nm (according to equation 2, Fig. 1b).

These results demonstrate that a majority of FDM minima in stimulated RBL cells generally co-localize with ER-tracker positive structures, which are barely detectable in resting cells. Importantly, distribution of the fluorescence ER-marker typically extends laterally beyond potential sub-plasmalemmal structures within the evanescent field and displays considerable inhomogeneity in fluorescence intensity. Hence, ER-PM junction morphology cannot be delineated by visualization of ER-marker alone in TIRFM. This is illustrated by a marked variation of ER-tracker intensity observed at constant GFP intensity levels in FDM minima (Fig. 3b), indicating general abundance of the ER-marker in junctional areas at highly variable intensities.

This set of results corroborates a substantial ER re-organization with a dramatic increase in sub-plasmlemmal, junctional ER in response to store-operated Ca^2+^ entry. A similar result was obtained with the genetically encoded ER marker, DsRed-ER (Suppl. Fig. 1). Reorganization of the ER was observed only in cells stimulated by the Ca^2+^ store depletion-reentry protocol but not in controls using vehicle (0.05% DMSO) instead of thapsigargin (data not shown).

Our results show a profound increase in ER-PM junction density and size in response to store depletion and indicate that the fluorescence intensity pattern of common ER-targeted fluorescent markers is spatially too heterogeneous to enable reliable monitoring of the ER-plasma membrane gap by TIRFM.

### YFP-MAPPER and FDM identify ER-PM junctions in TIRFM

In a further test, we expressed a fluorescent ER-targeted fusion protein that is able to bridge the ER-PM junctional distance by interaction with negatively charged lipids in the PM. The molecule, which accumulates and reports specifically junctional regions, has recently been introduced by the group of Jen Liou^7^ and termed MAPPER. This molecular reporter is comprised of a genetically engineered STIM1, fused in its N-terminus to a GFP and in which the coiled-coiled domains and the serin/prolin rich region in the cytosolic part of the protein were replaced by a spacer while the C-terminal polybasic domain was preserved to recognize PM lipids. Thus, MAPPER is able to bridge the distance between ER and PM without the need of any conformational changes or protein-protein interactions and is constitutively targeted to ER-PM junctions. We co-transfected RBL-2H3 cells with YFP-MAPPER and cytosolic mCherry as FDM reporter for a direct comparison of the localizations.

**Figure 4:**
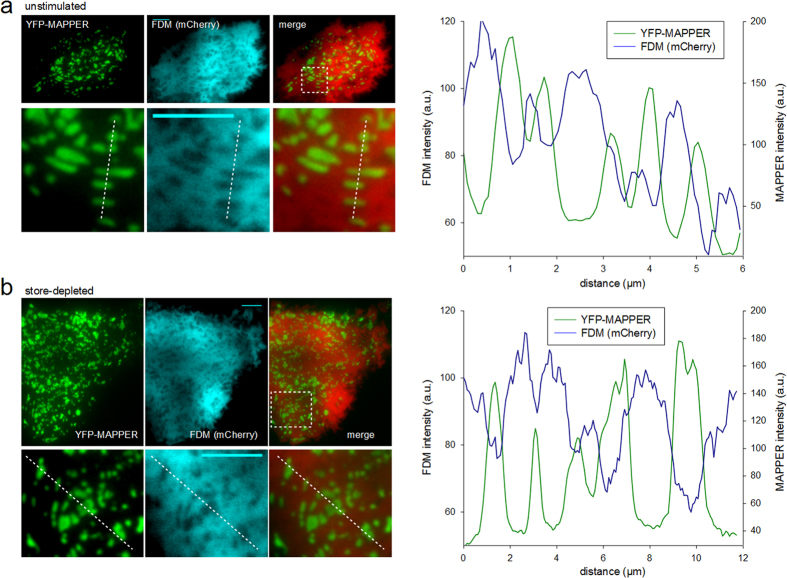
YFP-MAPPER and FDM identify ER-PM junctions in TIRFM. (**a**) Top, left: TIRFM micrographs of a non-store depleted RBL-2H3 cell, co-expressing YFP-MAPPER (green, left) and cytosolic mCherry (cyan, right). Below: Magnified details of area indicated in top panel including a color overlay (merge). Right: Intensity line-scans of YFP-MAPPER fluorescence (green) and FDM intensities (blue) as indicated by the dotted line in detail image. (**b**) Top, left: TIRFM micrographs of a store depleted RBL-2H3 cell, co-expressing YFP-MAPPER (green, left) and cytosolic mCherry (cyan, right). Below: Magnified details of area indicated in top panel, including a color overlay (merge). Right: Intensity line-scans of YFP-MAPPER fluorescence (green) and FDM intensities (blue) as indicated by the dotted line in detail image. Bars = 5 μm.

In both resting and SOCE-stimulated RBL-2H3 cells, YFP-MAPPER was targeted to form sub-plasmalemmal patches with a size, ranging from very small punctae like structures to large irregularly shaped junctional areas of several μm^2^ (mean = 0.56 ± 0.05 μm^2^) (Fig. 4). These MAPPER-positive junctional areas overlapped perfectly with FDM minima substantiating correct junction identification. Mean intensity in FDM minima was 56% ± 0.8% (S.E.M., N = 4 cells, n = 923 junctions), corresponding to a mean ER-PM distance of ~41 nm (according to equation 2, Fig. 1b). The inverse correlation between MAPPER and FDM reporter (mirror-image) convincingly demonstrates suitability of FDM to precisely report ER-PM junctions. More than 70% (non-stimulated: 79.7% ± 4.5 S.E.M., N = 6 cells, store depleted: 74.8 ± 7.4 S.E.M., N = 5 cells) of the junctions identified by MAPPER overlapped with a local minimum of FDM. Due high lateral blur in FDM, very small clusters of MAPPER (<0.3 μm^2^) could not be clearly resolved in the FDM images and these small structures are very likely the main reason for overlap values below 90% as the subpopulation of large structures showed a mean overlap of 91% (±4.37 S.E.M., N = 3 cells).

The use of MAPPER as a well-characterized direct reporter for ER-PM junctions confirmed high reliability of FDM for detection of ER-PM contact sites. Importantly, the observed high density and the relatively large size of junctions in non-stimulated, MAPPER-positive cells indicate that the membrane-bridging marker significantly promotes spontaneous junction formation.

### Monitoring STIM1-Orai1 competent ER-PM junctions by FDM

Besides MAPPER as a non-regulated junctional marker, we used also functional YFP-STIM1 to report SOCE-competent ER-PM contact sites. We observed a microtubule-associated, sub-plasmalemmal distribution of YFP-STIM1 fluorescence in resting cells along with distinct spherical and mostly mobile FDM minima lacking STIM1 accumulation (vesicles). After store depletion and SOCE, co-localization of immobile, irregularly shaped FDM minima with STIM1 clusters was observed, demonstrating reliability of FDM as a mean to detect STIM-Orai-competent junctions (Suppl. Fig. 2).

**Figure 5:**
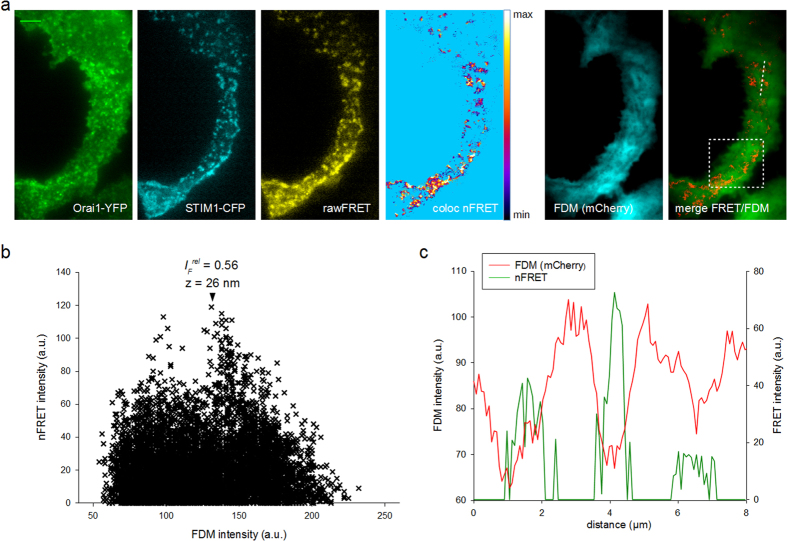
Correlation of local cellular FDM intensities with STIM1-Orai1 FRET. (**a**) TIRFM micrographs of a store-depleted RBL-2H3 cell, expressing Orai1-YFP (green), STIM1-CFP (cyan), Orai1-STIM1 raw FRET image (yellow), co-localized bleed-trough-corrected net FRET (coloc. nFRET), FDM (mCherry, cyan) and a color overlay image of nFRET (red) and FDM (green). Bar = 5 μm. (**b**) Relation between FDM- and nFRET-intensities within a homogenously illuminated cell area (indicated in a, right). High intensities are detected at lower FDM intensities, with a peak at 56% of the maximal intensity corresponding to an ER-PM distance of z ~26 nm after correction for residual background fluorescence (~15%). (**c**) Line-scans of FDM (red) and nFRET (green) from a section indicated in a, right.

To further test reliability of the FDM approach and to explore suitability of the method for live-cell monitoring of inter-membrane distance and nano-architecture within junctions, we applied FDM in combination with a common TIRF-FRET approach that reports membrane–bridging events by the STIM1-Orai1 machinery. The physical interaction of STIM1 and Orai1 takes place in junctions that have recently been characterized by TEM^12^,^13^. FRET intensity generated by interacting STIM1-CFP and Orai1-YFP fusion proteins is critically depending on the distance between the membranes hosting the fusion proteins, and can therefore be utilized not only to identify contacts but also as a readout reporting junctional distance. As shown in Fig. 5, FRET signals were clearly co-localized with FDM minima. Moreover, we indeed observed the expected inverse correlation between FRET- and FDM-intensity values in the range corresponding to inter-membrane distances of 26 to 80 nm. FRET efficiency showed a peak at 26 nm (FDM intensity of 56–15% residual background) and in most experiments declined at larger as well as smaller inter-membrane distances (Fig. 5b,c).

The observed biphasic relation was characterized by a drop in nFRET intensity at very low FDM intensities, corresponding to small (<20 nm) membrane gaps. This observation might be explained by the predicted weakening of STIM1-Orai interactions at sub-optimal small ER-PM distance^14^. Hence, our results suggest FDM as method that enables monitoring of ER-PM junctional morphology.

### Monitoring dynamics of ER-PM junctions by FDM

Our initial experiments to visualize ER-PM contacts in RBL mast cells provided evidence for profound reorganization in the cortical, sub-plasmalemmal ER in response to store depletion and SOCE. This prompted us to further analyze the dynamics of ER-PM junction architecture during SOCE. For this purpose we again combined FDM with heterologous expression and TIRFM of the functional reporter YFP-STIM1 to characterize the temporal relation between junctional architecture and activation of the SOCE pathway.

**Figure 6:**
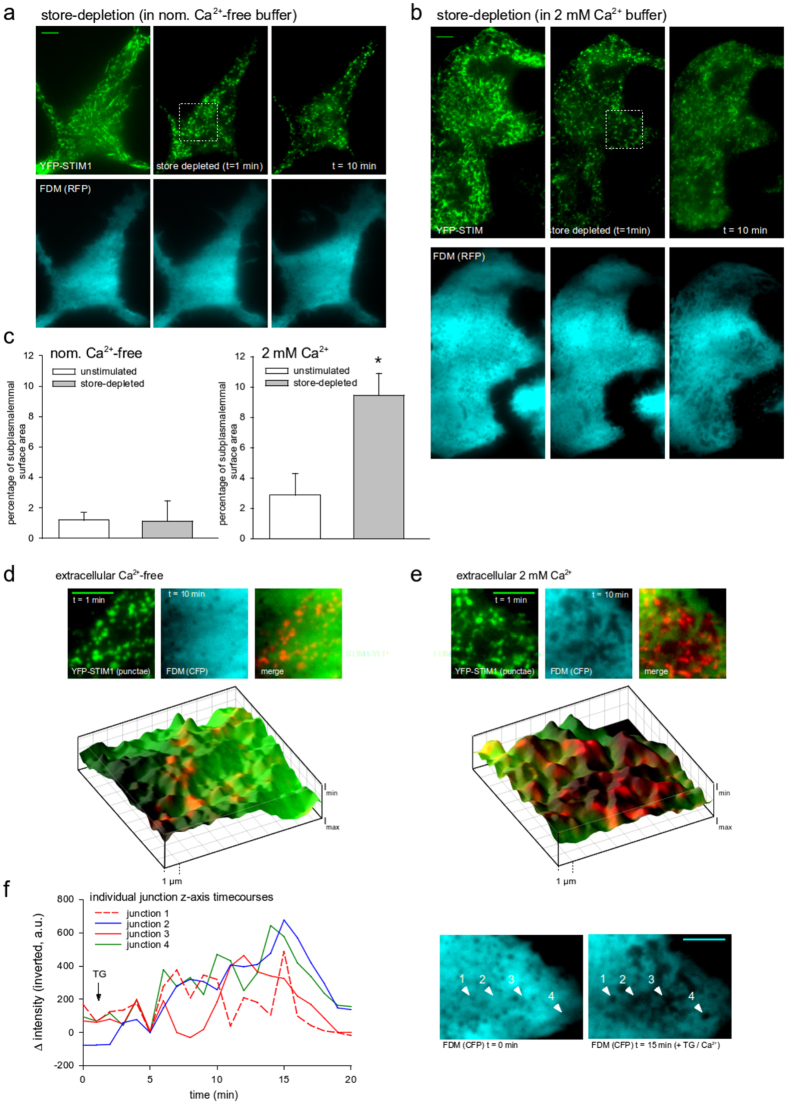
Formation of large junctional structures after stimulation with thapsigargin depends on extracellular Ca^2+^ in RBL-2H3 cells. (**a**) TIRFM micrographs of YFP-STIM1 (green, top) and FDM (mCherry) (cyan, bottom) in RBL-2H3 cells before (left), just after store depletion (center) and 10 min after stimulation (right) in absence of extracellular Ca^2+^. (**b**) YFP-STIM1 (green, top) and FDM (mCherry based) (cyan, bottom) of RBL-2H3 cells before (left), just after store depletion (center) and 10 min after stimulation (right) in presence of 2 mM extracellular Ca^2+^. (**c**) Mean percentage (±S.E.M., N = 5 cells) of TIRF junction surface area in unstimulated (white bar) and store-depleted RBL-2H3 cells (grey bar) in absence (left) or presence of 2 mM Ca^2+^. Asterisk indicates statistical significance (t-test). (**d**,**e**) Detail image sections as indicated in (**a**). Top: YFP-STIM1 punctae (t = 1 min, green), FDM junctions (t = 10 min, cyan) and color overlay (right) in absence of extracellular Ca^2+^. Bottom: 3D reconstruction of sub-plasmalemmal organelle surface within the evanescent field based on FDM and superimposed color merge image displaying localization of YFP-STIM1 clusters. I_min_ corresponds to position of the PM cytoplasmic surface. (**f**) Time-courses of inverted mean intensity in individual junctions of a single cell (relative to a non-junctional cytosolic reference point) in presence of 2 mM extracellular Ca^2+^. Store depletion is indicated by arrow. Right: Positions of junctions as indicated left before (t = 0 min) and after stimulation in presence of 2 mM extracellular Ca^2+^ (t = 15 min). Bars = 5 μm. Image RGB colors were set to cyan for better visibility of FDM structures.

Simultaneous monitoring of YFP-STIM1 clustering and FDM over a time period of >20 min, revealed that the fate of junctions was dependent on the absence or presence of extracellular Ca^2+^. Store depletion with thapsigargin (5 μM) in the absence of extracellular Ca^2+^ (nominally Ca^2+^-free) induced STIM1 punctae formation without a detectable remodeling and extension of individual junctional structures. As shown in Fig. 6a,b, the YFP-STIM1 punctae-pattern persisted over time in the absence of extracellular Ca^2+^, while store depletion in the presence of 2 mM Ca^2+^ and thus SOCE, induced punctae, which slowly dissipated within 20 min. By contrast, junctional areas identified by FDM enlarged progressively in the presence of extracellular Ca^2+^. Mean intensity in FDM minima was 61% ± 0.8% (S.E.M., N = 4 cells, n = 631 junctions) corresponding to an ER-PM distance of ~47 nm.

Overlay image analysis including an FDM based 3D-reconstruction of subcellular ER topology displaying YFP-STIM1 punctae immediately after store depletion (t = 1 min) and late FDM minima (t = 10 min) suggested that extended junctions originated from punctae structures and thus at positions of SOCE (Fig. 6d,e).

SOCE-dependent restructuring of nano-architecture was not dependent on STIM1- expression, as the phenomenon was similarly observed in RBL-2H3 cells expressing cytosolic CFP as FDM reporter only (Suppl. Fig. 3). Moreover, the observed increase in junctional surface area was not a response to irreversible SERCA inhibition and persistent ER Ca^2+^ depletion, as a similar enlargement of ER-PM junctional areas was observed when RBL cells were activated via transient store depletion by BHQ to allow ER refilling during SOCE (Suppl. Fig. 4).

As FDM allows for semi-quantitative characterization of inter-membrane distance at relatively high temporal resolution, we further tested whether junction enlargement is a continuous process leading to a stable, uniform inter-membrane gap. Figure 6f illustrates time-courses of inverted mean FDM intensities in individual junctions of a single cell after store depletion and in absence and presence of extracellular Ca^2+^. Mean FDM intensities of individual junctions in the presence of extracellular Ca^2+^ displayed considerable vertical oscillations during lateral extension, indicating large cyclic variation of inter-membrane distance at different positions within the cell. This was not observed in the small number of junctions detected in Ca^2+^-free conditions. Hence, we conclude that Ca^2+^ entry into the initially existing junctional space triggers highly dynamic, phasic changes in the architecture of SOCE–competent ER-PM junctions.

## Discussion

In this study, we introduce fluorescence density mapping (FDM) as a simple and versatile extension of TIRFM. FDM enables reliable detection of PM-near organelles localized within distances of ~10–200 nm from the PM cytoplasmic surface. Thus, FDM allows the visualization of nano-architecture in organelle plasma membrane junctions and to study architectural dynamics of any sub-plasmalemmal organelle.

### Monitoring lateral dimensions of ER-PM junctions by FDM

FDM is based on the principle of monitoring the displacement of a homogenously distributed fluorophore within evanescent field to quantitatively analyze subplasmalemmal organelle structure. The general visualization principle has previously been used to monitor vesicle fusion events^15^,^16^ but has so far not been utilized to quantitatively monitor nano-scale architecture of organelle. Our initial results obtained by a combined approach using FDM and different ER markers demonstrated that TIRFM-based visualization of organelle-targeted fluorescence markers was not suitable to estimate ER-PM distances and to precisely delineate junctional regions. This is mainly due to lack of internal reference for the maximal fluorescence intensity obtained when the distance to the PM approaches zero. By contrast, the drop in fluorescence intensity caused by a displacement of a homogenously distributed, cytosolic fluorophore from the evanescent field can be easily expressed relative to the maximum fluorescence at organelle-free positions and will approach zero when the organelle attaches to the PM. Consequently, lateral borders of junctional areas can be identified by FDM on the basis of a defined level of relative fluorescence reduction.

Our comparison of fluorescence distributions of ER-markers with FDM showed that the marker signal was accumulated, but not exclusively detectable within junctional areas. Experiments using ER-Tracker, a BODYPY-labeled blocker of ER potassium channels (glibenglamide), and alternatively the luminal ER-DsRed protein indicated that these ER-targeted fluorophores are not homogenously distributed within the organelle membrane but tend to cluster, while distribution of the FDM reporter was essentially homogenous within junctions and in organelle-free areas. This was convincingly demonstrated by continuous intensity levels within junctional and non-junctional regions defined by use of the bridging marker molecule (YFP-MAPPER). A significant overlap of FDM minima with YFP-MAPPER accumulation corroborated our conclusion that FDM enables visualization and spatial delineation of junctional areas at unprecedented precision in combination with essentially low interference with junctional structure and function. One limitation of FDM arises from lateral blur and a relatively low lateral (2D) resolution. Utilizing MAPPER as reference, the smallest detected FDM structures in this study were measured to have a diameter of ~0.3 μm. This resolution covers the typical STIM1-Orai1 punctae dimensions that have been reported to have a diameter of ~1 μm^17^. 2D-deconvolution techniques or selection of fluorophores with different properties and/or concentrations may be used to further optimize lateral resolution.

Lateral dimensions of junctions determined by FDM were variable from cell to cell, with maximal diameters ranging from 300 nm up to several μm. These dimensions are clearly larger than mean values obtained by TEM (mean ~200 nm). However, measurements based on TEM slices do not yield maximal diameters of the structures and mean values derived from single TEM images are thus expected to be smaller than the maximal diameters.

An important strength of FDM is the availability of additional depth (z) information, which can be utilized to delineate not only the PM surface shape of a defined set of junctions, but to reconstruct a topology of organelle (ER) surface landscape underneath the PM.

### Monitoring the junctional gap distance and 3D topology reconstruction of ER-PM contact sites

The relative z-axis resolution of FDM can be optimized by adjusting the penetration depth of the evanescent field via the TIRF angle. Although the typical cell-to-cell differences in fluorophore concentrations prevent an accurate determination of absolute distances from FDM intensities, estimation of the ER-PM gap dimension appears feasible based on the mathematical description (equation 2), which relates local fluorescence intensity to intermembrane distance at a given evanescent wave penetration depth. Validity of this approach was tested by use of different molecular rulers. FDM analysis of ER-PM junctions expressing YFP-MAPPER fluorescence or STIM1-Orai1 FRET yielded estimates of junctional intermembrane distances in the range of 37–47 nm. These values are slightly larger than those found in our TEM experiments. The reason for this deviation might be a contribution from background fluorescence of cytosolic areas below the ER (see Fig. 1a) to FDM intensities, which according to equation 1 and the measured (TEM) vertical dimensions of the ER, may amount to about 15% of FDM intensity. Considering this correction, FDM estimates of the junctional gap are in the range of 20–35 nm and thus in line with TEM reports.

Orai1-STIM1 interaction, as detected by local FRET of fusion proteins, showed a maximum at FDM- minima with an intensity drop of 44%. When correcting for residual background fluorescence (~15%) this value corresponds to a membrane gap of 26 nm, which is within the range of reported membrane distances for STIM1-Orai1 junctions^5^,^11^,^18^. Interestingly, we observed a slight decay of FRET efficiencies at very low distances/FDM intensities suggesting imperfect bridging or orientation of fluorophors. This is consistent with a recent report showing a decline in STIM1-Orai1 binding at close proximity of the junctional membranes^14^. Collectively, these data support the conclusion of FDM being a reliable technique for semi-quantitative evaluation of ER-PM distances and junctional architecture.

It is unclear at this point if FDM accurately resolves organelle topologies within a distance of <20 nm at the PM as such narrow junctions may limit free diffusion of fluorophors. The reported inter-membrane gap of ~20 nm in STIM-Orai competent junctions^5^,^11^,^14^ does not spatially exclude GFP, as its ß-barrel structure is about 4 nm long and 3 nm in diameter^19^. Likewise, GFP has been shown to diffuse freely in the ER-lumen, which in large parts resembles the narrow compartment of the junctional space^20^.

In aggregate, our results suggest FDM as a valuable method for visualization of organelle-PM contact sites and as a tool to reliably estimate the relative proximity of ER structures to the PM.

Resting cells typically displayed a significant density of PM-associated vesicles, which were discriminated from junctions by their motility, uniform spherical shape and lack of ER markers. YFP-STIM1 fluorescence condensed into punctae-like structures upon store depletion and punctae of larger dimensions (>0.3 μm^2^) clearly overlapped with FDM minima. Unlike in YFP-STIM1-expressing cells, density of junctional areas in YFP-MAPPER expressing cells did not depend on store-depletion. MAPPER-positive FDM minima were observed in resting as well as in store depleted cells. The high density of junctional areas in resting, MAPPER-YFP expressing RBL-2H3 cells, suggests that MAPPER facilitates formation of junctional areas. Albeit the initial functional characterization of MAPPER indicated lack of interference with store-operated Ca^2+^ signaling^7^, the here observed promotion of junctional structures in resting cells is likely to interfere with other cell functions linked to junctional architecture.

While heterologous expression of MAPPER and, to a lower extend, also STIM1 facilitated the formation of ER-PM junctions, (over-)expression of these proteins was not required to induce junctions. This conclusion was corroborated in RBL-2H3 cells expressing CFP as FDM reporter only.

Having a method at hand that provides information about the 3D-topology of junctional surfaces at a high temporal resolution, we set out to characterize this dynamic feature of STIM-Orai competent junctions in more detail.

One unexpected finding of our investigation of STIM-Orai-competent junctions was the robust increase in number and size of ER-PM junctional structures in RBL-2H3 mast cells, which was clearly extracellular Ca^2+^ dependent and occurred with a striking delay after SOCE. Enlargement of ER-PM junctional area in response to STIM/Orai (SOC) activation has previously been reported^12^ and a more recent study demonstrates Ca^2+^-mediated extension of junctional ER^13^. To our knowledge, we provide here the first demonstration of Ca^2+^ entry-triggered extension of ER-PM junctions that persist after dissociation of STIM-Orai punctae and termination of SOCE.

### STIM1-Orai1 induced ER-PM junctions extend in a dynamic manner upon store-operated Ca^2+^ entry

Our initial TEM analysis demonstrates that density and distribution cortical ER is significanly different in basal and store-depleted RBL-2H3 cells. After store depletion, we found abundance of ER structures in close proximity of the PM, displaying parallel membrane alignment within distances of ~15–20 nm, a range proposed for STIM1-Orai1-competent junctions^5^,^14^,^21^. The phenomenon of ER translocation during SOC activation and the formation of differently structured cortical ER elements was previously observed by electron microscopy in HeLa cells^13^. Consistently, FDM detected the generation of junctional regions in response to store depletion of RBL-2H3 mast cells. The recently proposed mechanism of punctae formation includes diffusion trapping of conformationally altered STIM proteins in pre-existing junctional areas of the PM as an initial step^5^. These pre-existing junctions were not clearly resolved by FDM, indicating these preformed junctions are rather small in lateral extension. Based on our results from TEM and FDM data, it appears rather unlikely that portions of voluminous cortical ER, which are low abundant in resting cells, contribute to pre-existing junctions providing a substrate for diffusion trapping. Thus, initial punctae formation might start at cortical ER sections that are very limited in lateral extension and volume and, as indicated by common TIRFM, appears to be initially tightly associated to the cytoskeleton^22^,^23^,^24^.

Asanov *et al.* reported that STIM1 complexes include microtubular endbinding protein 1 (EB1) at rest and rearrange after store-depletion to associate with binding protein adenomatous polyposis coli (APC)^22^. Such changes in STIM1 binding partner proteins at a critical step of SOCE may be the basis of fast ER remodeling that is ultimately affecting STIM1 distribution as well as ER volume and structure.

As anticipated, newly formed STIM punctae showed a clear overlap with FDM minima, consistent with localization within junctional ER. Starting with this initial event in the signal transduction process, the fate of ER-PM junctions in stimulated RBL-2H3 cells was found to strictly depend on the entry of extracellular Ca^2+^. Image overlay based analysis showed that late junction areas overlapped with initial punctae positions in presence of extracellular Ca^2+^ (Fig. 6e,f). Taken together, our observations indicate a mechanism of ER-PM surface enlargement initiated by Ca^2+^-entry trough STIM-Orai complexes and based on membrane bridging by Ca^2+^-dependent PM-ER tethering protein(s). According to our results, this secondary tethering occurs with a significant delay and clearly outlasts the clustering of STIM1 in the ER of mast cells. Such a process might serve as an adaptive response of the cell with the aim to increase the efficiency of ER Ca^2+^-uptake. Nonetheless, this process did not depend on persistent inhibition of SERCA by thapsigargin, as transient store depletion by BHQ initiated similar rearrangement of sub-plasmalemmal structures.

Currently, we can only speculate about the molecular processes contributing to such a Ca^2+^-dependent remodeling of junctional ER. Attractive candidates are proteins of the synaptotagmin family which are already known to play important roles in mast cell degranulation and exocytosis^25^,^26^,^27^. As synaptotagmins are Ca^2+^-dependent proteins that stabilize junctions in order to enable trans-compartimental transport of lipids^28^, they might contribute to the observed Ca^2+^-dependent increase in STIM-Orai induced ER-PM contact area. This view is also in agreement with a report by Maleth *et al.*, showing the tethering of E-Syt1 to STIM1-Orai1 complexes^29^.

In aggregate, we developed and characterized a novel method for topology reconstruction of ER-PM junctions. This approach enables insight into nano-scale architectural dynamics in organelle-plasma membrane communication.

## Materials and Methods

### Fluorescence labeled protein constructs and dyes

Human C-terminally ECFP-tagged STIM1, N-terminally EYFP-tagged STIM1 (STIM1; accession number NM_003156) and C-terminally EYFP-tagged Orai1 (accession number NM_032790) were kindly provided by C. Romanin, Linz, Austria.

YFP-MAPPER (membrane attached peripheral ER) was kindly provided by the group of Dr. J. Liou, University of Texas Southwestern Medical Center, US and has been produced by replacing the mCherry-STIM portion of SP-mCherry-STIM construct with the sequences of YFP, the transmembrane domain of STIM1, FKBP12-rapamycin flanked by two helical linker sequences (EAAAR) and a polybasic tail sequence taken from Ras-like protein Rit.

For cytosolic expression of YFP, CFP and mCherry, as required for fluorescence density mapping, cells were transfected with pEYFP-C1, pECFP-C1 or pCMV-mCherry vectors.

Fluorescence labeling of the ER was performed using an ER-Tracker Red (glibenglamide BODIPY^®^) dye (Molecular Probes, Mountain View, US). Cells were incubated for 30 min at 37 °C with ER-Tracker Red in glass bottom dishes and used for microscopy after exchange of the incubation medium for experimental buffer solution. Alternatively, a fluorophore conjugated ER-marker protein pDsRed2-ER (Clonetech, Mountain View, US) was expressed in RBL-cells. This protein represents a fusion construct of the ER targeting sequence of calreticulin, the ER retention sequence KDEL and a RFP-tag.

### Cells and heterologous transfection

RBL-2H3 cells (ATCC, CRL-2256) were cultivated in high glucose containing DMEM with 10% FCS at 37 °C and 5% CO_2_ and in presence of 100 IU penicillin and 100 μg/ml streptomycin. Transfections were performed by electroporation using a GenePulser II (BioRad, Hercules, US) with 300 V and 275 μF using 4 mm slit cuvettes and a total of 20 μg DNA per cuvette (400 μl cell suspension). RBL-2H3 cell suspension was obtained by trypsinizing the adherent cells for 5 min at 37 °C and subsequent centrifugation at 900 rpm. The pellet was re-suspended in 400 μl of penicillin/streptomycin-free medium. Cells were seeded on glass-bottom dishes and used 24 h after electroporation.

### Buffer solutions and stimulation

Standard experimental buffer solutions contained (in mM): 140 NaCl, 5.4 KCl, 1 MgCl_2_, 10 glucose monohydrate, 10 HEPES, ±2 CaCl_2_, ph7.4. Cells were store depleted by 5 μm thapsigargin, by adding 1 μl of a 10 mM stock solution in DMSO to 2 ml buffer or using 15 μm 2.5-di-t-butyl-1,4-benzohydroquinone (BHQ), by adding 2 μl of a 15 mM stock solution in EtOH to 2 ml buffer.

### Transmission electron microscopy

RBL-Cells were grown on an Aclar film (Gröpl, Tulln, Austria), fixed in 2.5% (wt/vol) glutardialdehyde and 2% (wt/vol) paraformaldehyde in 0.1 M cacodylate-buffer for 45 min, postfixed in a mixture of 1% (wt/vol) osmium tetroxide and 1% potassiumhexacyanoferrate (wt/vol) (aqueous) for 45 min, dehydrated in graded series of acetone and embedded in TAAB epoxy resin (Agar Scientific, Essex, GB). Ultrathin sections (70 nm) were cut with a UC6 Ultramicrotome (Leica Microsystems, Vienna, Austria). Sections were stained with platinum blue for 15 min and lead citrate for 7 min. Investigation of the ultrathin sections were performed using a Tecnai G2 20 transmission electron microscope (FEI, Eindhoven, Netherlands) with a Gatan Ultrascan 1000 charge coupled device (CCD) camera at an acceleration voltage of 120 kV.

### Total internal reflection microscopy

TIRF microscopy was performed on an Observer2D microscope (Zeiss, Jena, Germany) equipped with an Orca2D camera (Hamamatsu Photonics, Hamamatsu, Japan) and a Visitron TIRFM system (Visitron Systems, Puchheim, Germany) equipped with a 100× oil immersion Zeiss TIRFM Objective. Excitation light was provided by 445 nm, 488 nm and a 561 nm diode laser lines (Visitron). For comparisons between fluorescence channels the focal plane for the images was kept constant. Wavelength induced differences in focal planes were compensated by the Orca2D camera (dual chip system).

### Image and FRET analysis

Image analysis was performed using ImageJ software (Rasband, W.S., ImageJ, U. S. National Institutes of Health, Bethesda, Maryland, USA, http://imagej.nih.gov/ij/, 1997–2016).

For quantification of junctional area sizes a local threshold (radius 20 pixel) was applied to the image to detect the local FDM minima. After inversion, the detected minima were converted into ROIs using the ImageJ “nucleus counter” plugin and ROI areas measured after spatial calibration.

ER-PM distance was calculated from FDM recordings by measuring the relative intensity difference between junctional areas and close non-junctional regions of a cell at a given evanescent field penetration depth (TIRFM angle). Assuming a maximal local fluorescence in the non-junctional region (I_max_), the relative intensity drop in the junction reflects (I_jun_/I_max_) the distance of the ER (z) in relation to the total penetration depth (d_p_) according to equation 2.

FRET analysis was performed with the ImageJ FRET analyzer plugin following the sensitized emission method for determining FRET (FRET = rawFRET − (C_Donor_ * I_Donor_) − (C_Acceptor _* I_Acceptor_), C = bleed-trough correction factors as determined by separate experiments using only one fluorophore).

### Theoretical base of the estimation of ER-PM distance by FDM

In TIRFM, cellular fluorophores are excited by an evanescent field that decays exponentially within a limited layer adjacent to the surface adherent PM area. This reduces background fluorescence and enhances resolution for observations in the surface attached portions of the membrane. In common practice, the excitatory penetration depth of the evanescent wave is adjusted within certain limits to levels between 50 nm and 300 nm by adjusting the incident angle of excitatory laser beam. The penetration depth (d_p_) of the evanescent wave depends on the TIRFM angle^30^ as described in:


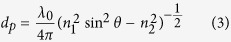


where *λ*_*0*_ represents the wavelength of the excitation light in the vacuum, *θ* is the angle to the normal at which the laser is incident on the surface, *n*_*1*_ is the refractive index of the glass and *n*_*2*_ is the refractive index of the specimen. The depth dependency of the excitation intensity due to the evanescent field is given by:


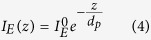


where *I*_*E*_ represents the depth dependent excitation intensity, *z* is the distance to the surface of total reflection, 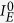 the intensity of the evanescent field at *z* = 0 and *d*_*p*_ the penetration depth of the evanescent wave. Accordingly, the evanescent wave excites fluorophores, with intensities strictly dependent on their distance to the surface of total reflection (coverslip/plasmamembrane contact area).

In our approach, we utilize GFP variants, which display a fairly homogenous distribution within the cytoplasmic evanescent field^31^,^32^. Based on this assumption, the overall emission intensity can be defined, according to Beer’s law, as a function of the thickness of the excited fluorophore containing layer (*z*):


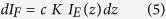


where dI_F_ is the fluorescence emission intensity of a layer with the thickness dz (infinitesimal small), c is the concentration of the fluorophore and K is a constant factor describing the relationship between excitation and emission intensity. The overall fluorescence emission intensity I_F_ of a confined layer is given by integration of equation 2 + 3 and results in:


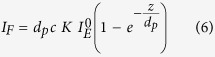


By omitting (mostly unknown) parameters determining absolute intensities (multiplicators d_p_, c, K and 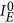) in this equation and thereby isolating the relative drop of intensity in ER-PM junction areas, we obtain equations 1 + 2.

## Additional Information

**How to cite this article**: Poteser, M. *et al.* Live-cell imaging of ER-PM contact architecture by a novel TIRFM approach reveals extension of junctions in response to store-operated Ca^2+^-entry. *Sci. Rep.* **6**, 35656; doi: 10.1038/srep35656 (2016).

## Supplementary Material

Supplementary Information
