# Supplementary material for: Live-cell imaging of ER-PM contact architecture by a novel TIRFM approach reveals extension of junctions in response to store-operated Ca2+-entry
Source: Sci Rep. 2016 Oct 19;6:35656. doi: 10.1038/srep35656 (PMC5069484; doi:10.1038/srep35656)
Supplement: Supplementary Information [file srep35656-s1.doc]

**Supporting Materials**

Live-cell imaging of ER-PM contact architecture by a novel TIRFM approach reveals extension of junctions in response to store-operated Ca^2+^-entry.

**Authors:** Michael Poteser^1*^, Gerd Leitinger^2^, Elisabeth Pritz^2^, Dieter Platzer^1^, Irene Frischauf^3^, Christoph Romanin^3^ and Klaus Groschner^1*^

^1^ Institute of Biophysics, Medical University of Graz, Harrachgasse 21/4, 8010 Graz, Austria

^2^ Institute of Histology, Research Unit “Electron Microscopic Techniques”, Medical University of Graz, Harrachgasse 21/4, 8010 Graz, Austria

^3^ Institute of Biophysics, Johannes Kepler University of Linz, Gruberstrasse 40, 4020 Linz

*Corresponding authors: [michael.poteser@medunigraz.at](mailto:michael.poteser@medunigraz.at), [klaus.groschner@medunigraz.at](mailto:klaus.groschner@medunigraz.at); Phone +43 316-380-4137, Fax +43 316-380-9660


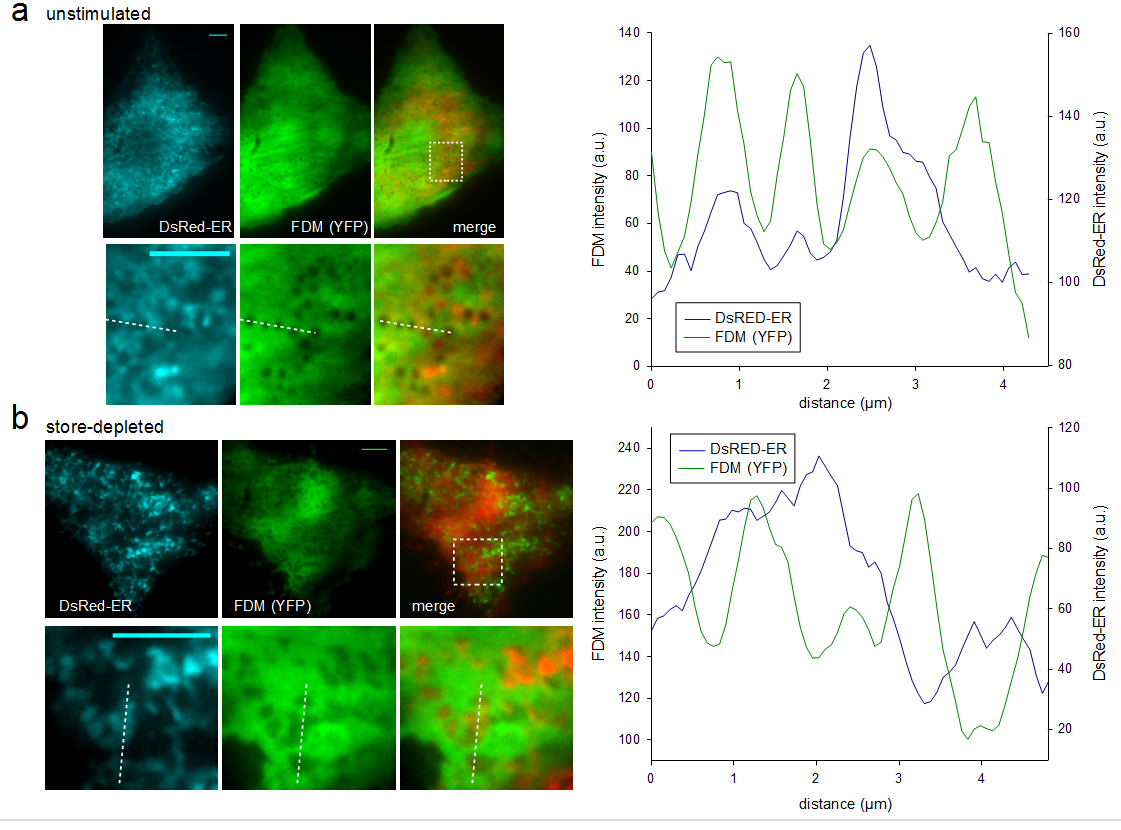


**Supplemental Figure 1: Store-depletion induced ER-PM junctions visualized by FDM and DsRed-ER.**

(**a**) Left, top: TIRFM micrographs of a non-stimulated RBL-2H3 cell expressing DsRed-ER (cyan) and cytosolic YFP (FDM, green) and a color overlay image (merge). Below: Detail images from area indicated in top image as indicated by white rectangle. Right: Line-scans of fluorescence intensity from a line as indicated in detail view (FDM: green, DsRed-ER: blue). (**b**) Same configuration as shown in a, but shortly (2 min) after store-depletion of RBL-2H3 cells. Bars: 5 µm.


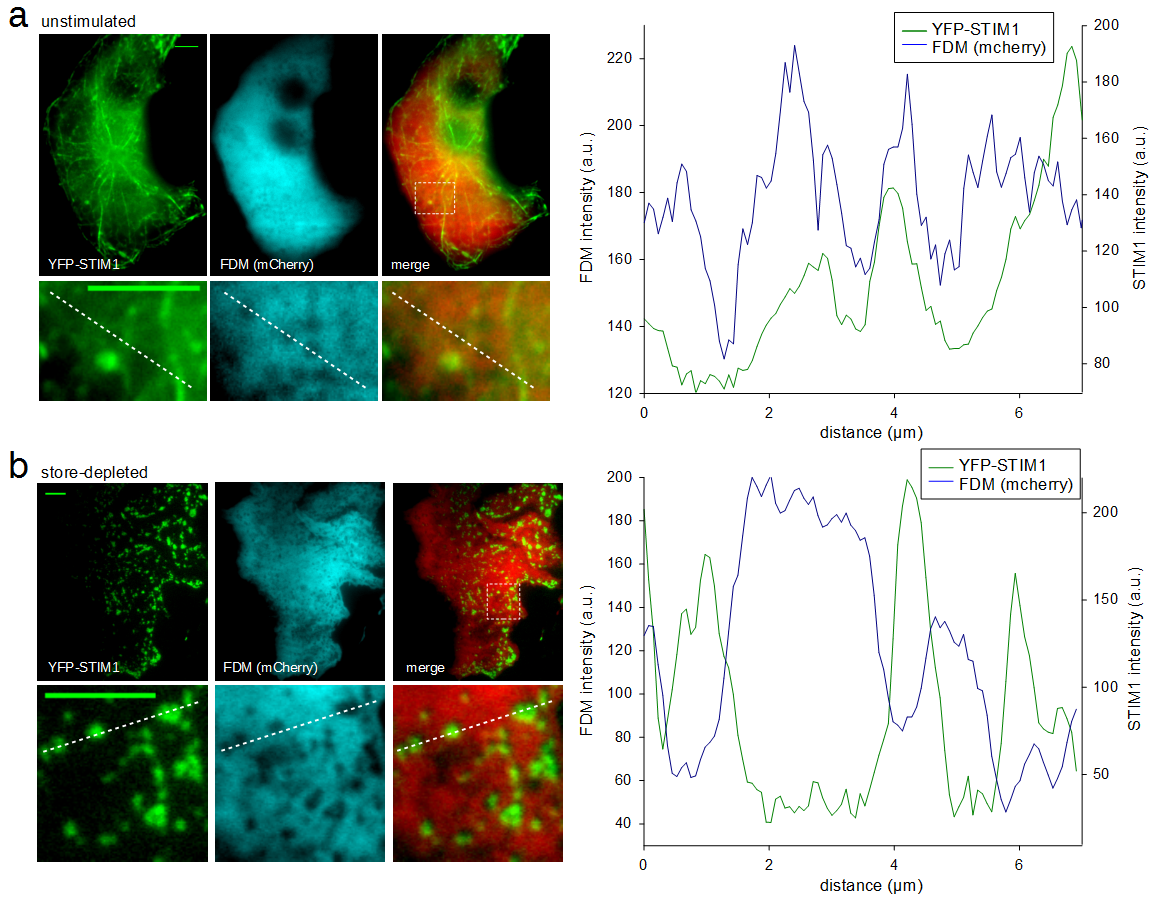


**Supplemental Figure 2: Store-depletion induced ER-PM junctions visualized by FDM and STIM1-YFP.**

(**a**) Left, top: TIRFM micrographs of a non-stimulated RBL-2H3 cell expressing STIM1-YFP (green) and cytosolic mCherry (FDM, cyan) and color overlay image (merge). Below: Detail images from area indicated in top image. Right: Line-scans of fluorescence intensity from a line as indicated in detail view (FDM: blue, STIM1-YFP: green). Note that structures detected by FDM in unstimulated cells mainly represent sub-plasmalemmal vesicles. (**b**) Same configuration as shown in a, but shortly (2 min) after store-depletion of RBL-2H3 cells. Bars: 5 µm.


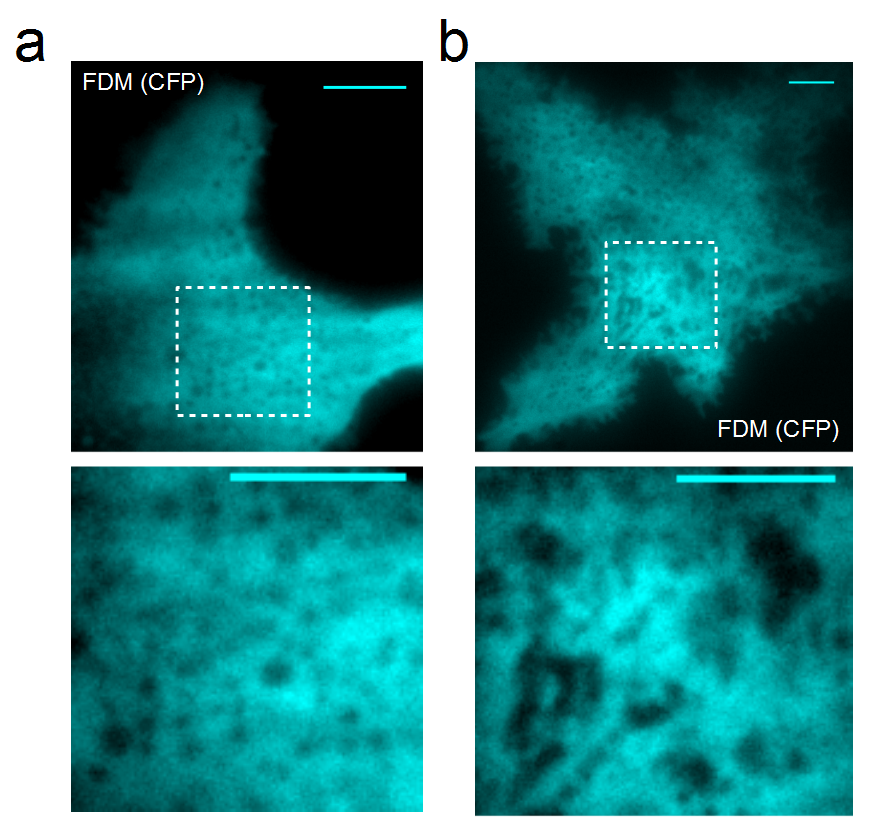


**Supplemental Figure 3: Large ER-PM junctions as identified by FDM are not dependent on MAPPER or STIM1 (over-)expression.**

FDM of a non-stimulated (**a**) and a store depleted (**b**) RBL-2H3 cell. Bottom: Detail view from cells shown above. Bars = 5 µm.


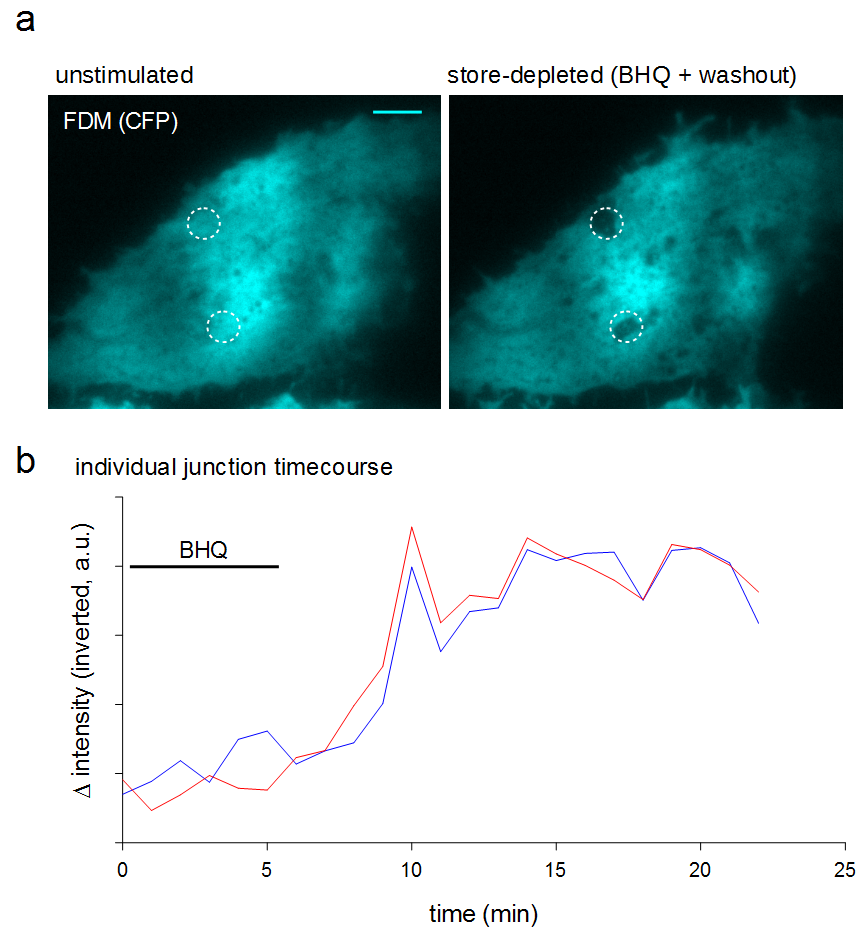


**Supplemental Figure 4: Transient inhibition of SERCA is sufficient to induce formation of junctions in RBL-2H3 cells.**

(**a**) FDM of a RBL-2H3 cell before (left) and after store depletion (right) in presence of 2 mM extracellular Ca^2+^. (**b**) Time-course of the inverted mean intensity (relative to a non-junctional cytosolic reference point) of two areas (as indicated in a, right) by 15 µm BHQ and subsequent washout of the SERCA inhibitor. Formation of junctions continues after washout of BHQ (t = 5 min).
